# The analysis of clinical and laboratory data: a large outbreak of dengue fever in Chaozhou, Guangdong province, China

**DOI:** 10.1007/s00705-019-04266-1

**Published:** 2019-05-17

**Authors:** Fen Lin, Hui Yang, Lin Zhang, Sen-Hai Fang, Xiao-Fen Zhan, Li-Ye Yang

**Affiliations:** 1 Central Laboratory, Chaozhou Central Hospital Affiliated to Southern Medical University, Chaozhou, 521021 Guangdong China; 2 Department of Laboratory Medicine, School of Medicine, Yangtze University, Jingzhou, Hubei China

## Abstract

A large-scale dengue fever (DF) outbreak occurred in Chaozhou, Guangdong province, China 2015. In our study, 528 dengue-positive patient samples were collected for clinical and laboratory data analysis. 491 cases (93.0%) were primary dengue fever (PDF), 22 cases (4.2%) were dengue hemorrhagic fever (DHF) and 15 cases (2.8%) were diagnosed with severe dengue fever (SDF). All cases were infected by dengue virus serotype 2 (DENV-2), and the isolated strains belonged to cosmopolitan genotype, which were grouped closely with Malaysia strains from 2010 to 2014. Moreover, the study showed that laboratory indices have significantly difference in PDF, DHF and SDF patients. A comprehensive analysis of these data could assist and guide the clinical diagnosis for DF, which has an important significance for the control of dengue virus infection.

## Introduction

DF known as break borne fever, is a mosquito-borne infectious tropical disease caused by DENV and the infection in humans results in different clinical manifestation, ranging from mild symptoms such as fever to life-threatening dengue hemorrhagic fever (DHF) and dengue shock syndrome (DSS). It occurs primarily in the equatorial regions of Africa, Latin Americas, South-East Asia, and the Western Pacific [1]. The incidence of DENV infection has increased 30-fold since the 1960s [2]. World Health Organization (WHO) estimates that approximately 3900 million people in 128 countries are at risk of infection with DENV [1, 3].

Guangdong was the most severe DF epidemic region in mainland China [4]. Chaozhou is an eastern city of Guangdong province, bordering Fujian province and the South China Sea, characterized by a humid subtropical climate, where the Aedes mosquitos are widely distributed and breed all year round. Dengue epidemics in Chaozhou have been reported sequentially since 1978, but there has been no massive outbreak, and only sporadic cases were reported in the past decade [5, 6]. In 2015, a large-scale DF outbreak occurred with 1372 confirmed DENV infections. The aim of the present study was to describe the clinical and laboratory data of hospitalized DF patients, and to determine the source of the virus causing this outbreak. Moreover, our study analysis highlighted the difference of laboratory indices in PDF, DHF and SDF patients.

## Materials and methods

A total of 528 dengue cases were collected from Chaozhou Central Hospital during September to October 2015. These subjects included 260 males and 268 females with a mean age of 44.17 years (range: 7 months to 95 years). DF diagnosis and classification of dengue severity was based on “national diagnostic criteria and principle of management of dengue fever (WS 216–2008)” [7] and the “Guangdong Province guidelines for dengue fever diagnosis and treatment (2014)”, patients were defined as DHF if they had fever, thrombocytopenia, bleeding, and evidence of plasma leakage, SDF patients were defined by evidence of plasma leakage associated with shock or respiratory distress, severe bleeding, or severe organ involvement. Clinical information (age, gender, sign, symptom) and laboratory data (complete blood counts, blood chemistry, virology) of DF patients were obtained by hospital information system LIS. All laboratory-confirmed dengue infected sera were stored at −80 °C for molecular analysis.

Viral RNA was extracted directly from the patients’ serum using QIAamp Viral RNA Mini kit (Qiagen) following the manufacturer’s instructions and stored at -70 °C for DENV typing and sequencing.

The serotypes including DENV-1, DENV-2, DENV-3 and DENV-4 were identified by reverse transcription polymerase chain reaction (RT-PCR) using the primers as described previously [8]. Briefly, a cDNA copy of a portion of the viral genome was produced in a reverse transcriptase reaction using SuperScript II Reverse Transcriptase (Invitrogen, USA). Then, consensus primers were used to amplify a 511-bp product with 2×Taq PCR MasterMix kit (Aidlab, Beijing, China) in the first round PCR. Nested PCR was performed with type-specific primers in the second round PCR.

40 serum samples were randomly selected for phylogenetic and evolutionary analysis from antigen positive serums. cDNA was synthesized by reverse transcription with SuperScript II Reverse Transcriptase (Invitrogen, USA). Two pairs of primers were designed to amplify the full length E gene: D2E1F:(5’-GAGCCCTGATTTTCATCTTACT-3’), D2E1R (5’-GCACTCTGATAACTATTGTTCCAT-3’), D2E2F (5’-GCGAAGAAACAGGATGTTGTTG-3) and D2E2R (5’-TTGAAGGGGATTCTGGTTGGA-3’). PCR products were confirmed by electrophoresis and purified using an Agarose Gel DNA Extraction kit (TaKaRa). Sequencing was then performed by the Invitrogen Company (Guangzhou, China). The nucleotide sequences of the viruses were analyzed to determine the sequence identity using the Lasergene software package (DNASTAR, Madison, WI).

The multiple nucleotide sequence alignments were performed using CLUSTAL W software (http://www.ebi.ac.uk/clustalw/). A total of 40 reference sequences of the DENV envelope gene with sampling dates were retrieved from GenBank. These sequences represented different geographical areas. Phylogenetic analyses was performed using MEGA 6.0 software. For the construction of phylogenetic trees, Tajima-Nei model with the maximum likelihood (ML) method was used. The statistical robustness and reliability of the branching order within each phylogenetic tree were evaluated by a bootstrap analysis with 1000 replications. D1/USA/Hawaii /1945 strain (AF425619) was used as out-group to root the tree.

Statistical analysis was conducted with SPSS 17.0 statistical software. Chi-square test was used for counting material, and t-test was used for measurement material, laboratory data were compared between the groups using single factor ANOVA analysis, P<0.05 was considered statistically different.

## Results

Based on the data from Chaozhou Center for Diseases Control (CDC), the first dengue case was confirmed in June 20, and all dengue cases were reported in Chaozhou until November 18, 2015. The majority of cases of DENV infection occurred in September and October, with daily newly emerged cases peaking in mid-September and more than 50 newly cases reported each day.

528 (male: 260, female: 268) clinically diagnosed and laboratory-confirmed DF cases were sampled from Chaozhou Central Hospital. Of these, 491 (93.0%) manifested PDF, 22 (4.2%) DHF, and 15 (2.8%) were SDF. The symptom composition was as follows: myalgia (362, 68.6%), headache (272, 51.5%), rash (141, 26.7%), bleeding (22, 4.2%), vomiting (41, 7.8%), diarrhea (38, 7.2%), ecchymosis (12, 2.3%). In addition, 25 (4.7%) cases required blood transfusion. 9 of the 15 SDF patients were elderly (>75 years) with underlying diseases-diabetes, coronary heart disease, and pulmonary tuberculosis. One case evolved to death and the other 14 cases eventually recovered and were discharged from hospital.

Among the laboratory-confirmed cases, approximately (433) 82% cases presented thrombocytopenia, (216) 40.9% exhibited leucopenia, (267) 50.6% and (229) 43.7% showed serum AST and LDH elevation, respectively. Compared to the PDF, PLT in patients with SDF was significantly lower than that of PDF patients. The laboratory indices also showed that the levels of WBC, ALT, AST, LDH, CK, CK-MB were significantly higher in SDF patients than those of PDF and PHF patients (P<0.05) (Table 1).

**Table 1:** Comparsion of laboratory indices among SDF and PDF, DHF groups

Index	PDF	DHF	SDF
WBC (10^9^/L)	3.72±0.091	3.22±0.241	5.41±1.100^★▲^
PLT (g/L)	81±2.307	39±6.584	19±5.037 ^★^
HCT (male)	0.385±0.003	0.397±0.007	0.380±0.022
HCT (female)	0.386±0.003	0.386±0.010	0.409±0.016
CRP (mg/L)	16±1.248	11±2.325	22±1.170
ALT (U/L)	39±1.745	43±11.921	85±28.648^★▲^
AST (U/L)	52±2.439	59±16.950	188±65.812^★▲^
TG (mmol/L)	1.46±0.052	1.67±0.291	1.82±0.360
CHO (mmol/L)	3.83±0.046	3.76±0.191	3.93±0.217
HDLC (mmol/L)	1.17±0.015	1.18±0.072	1.09±0.077
LDLC (mmol/L)	2.04±0.038	1.89±0.180	2.02±0.214
CK (U/L)	262±26.165	128±20.057	800±248.577^★▲^
CK-MB (U/L)	16.89±0.378	16.48±1.466	22.79±3.22^★▲^
LDH (U/L)	268±6.273	276±23.117	485±126.123^★▲^

★: severe dengue versus primary dengue, P<0.05

▲: severe dengue versus dengue hemorrhagic fever, P<0.05

The E gene from the serum samples of 40 patients with DENV-2 virus infection were amplified and sequenced. All the 40 DENV-2 isolates sequenced in this study revealed 96.4–98.2% nucleotide sequence similarity among them.

**Fig. 1:**
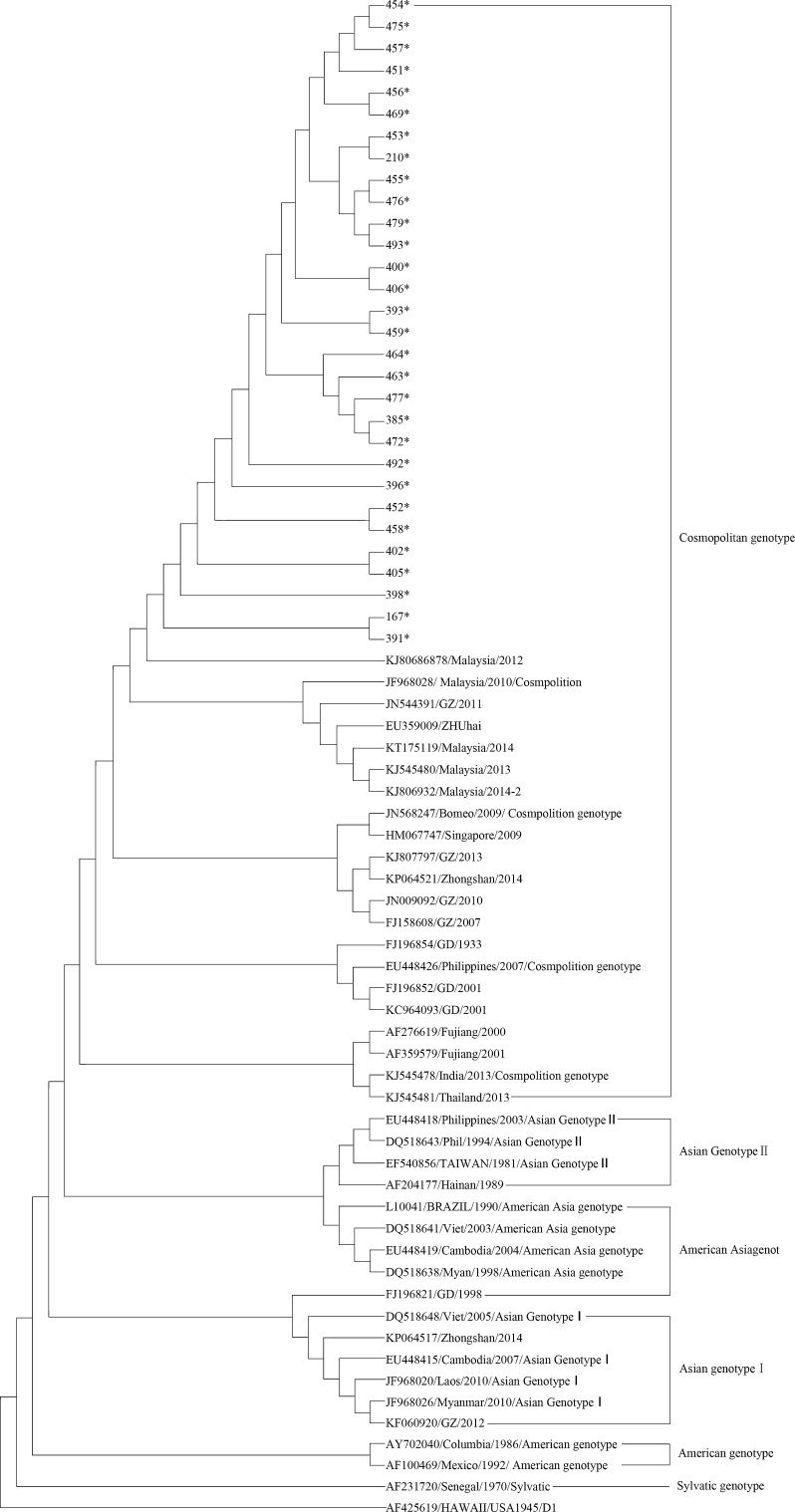
Phylogenetic tree of DENV-2. The DENV-2 isolated in this study were marked with asterisk (*)

Phylogenetic analysis was performed using sequences of DENV-2 E gene and 40 reference strains from global available in Genbank. As shown in Figure 1, the phylogenetic tree demonstrated that all the strains from this study were clustered into cosmopolitan genotype, which were closely related to DENV-2 isolates previously circulating in southeast Asia. Sequence alignment showed that the E protein of newly isolated DENV-2 strain had 100% amino acid homology to strains isolated from Malaysia in 2012, in contrast to the strains observed in 2014 Guangdong epidemic.

Compared to other strains isolated in Guangdong province of China, the E protein of the strains isolates in this outbreak share above 99.8% amino acid homology to recent Guangdong DENV-2 isolates since 2001. This result demonstrates that the newly isolated DENV is not “complete novel”, the virus was present for a long time.

## Discussion

Historically, DF has re-emerged in the Southern provinces of China. Chaozhou, belonging to Chaoshan region of Guangdong, it is well known as the hometown of Chinese and Ceramic Capital of China. Chaozhou has frequent interactions with many East Asia countries that have DENV epidemics. In 2015, hot weather and rain prevailed in Chaozhou, and mosquitoes multiplied wildly, which contributed to the spread of dengue virus. During September to October, 1372 dengue cases were reported in this city, and 528 confirmed DENV infections were collected from our hospital for clinical and laboratory data analysis.

Majority of the patients admitted to our hospital showed typical but mild dengue fever. There were 15 patients who developed SDF, most of them were elderly (>75 years) and had underlying diseases like diabetes. The reason why only few DENV infected individuals progressed to severe dengue disease is poorly understood.

As expected, PLT in patients with SDF was significantly lower than that of PDF and DHF patients. Moreover, DHF/DSS with prolong and profound shock and organ failure, especially liver failure were commonly observed. This was confirmed in this study by higher degree of ALT, AST elevation and CK, LDH values in DF patients, and was pronounced in SDF where it reached a 3~4-fold increase. This observation was in accordance with many previous studies [9, 10]. Our data indicated that elevated AST and ALT occurred in 50.6% and 29.9% of DF patients, respectively. AST is not only expressed in liver but also in the heart, skeletal muscle, brain, and kidneys. DF infection can cause acute damage to these organs and raised AST levels may not only be due to liver involvement. One study in Thailand found DF may be an important cause of acute liver failure in children [11]. Since the majority of our patients were adults, additional studies in pediatric populations will be useful for confirming this observation

Previously, the study by van Gorp et al. reported higher serum TG levels and lower serum CHO, HDL and LDL levels in SDF disease, which suggested that these levels could be used as prognostic markers to predict clinical outcome [12]. The reason for these lipoproteins changes in dengue infection is unclear. *In vitro* studies of the pathophysiology of DENV and other flavivirus infections suggest that lipids and lipoproteins may play a role in modifying virus infectivity in target cells [13]. In our study, we found no association between CHO level and dengue severity. In the two previous studies that used multivariable models for examining the relationship between CHO and SDF, HDL-C, LDL-C and SDF outcome, a link was observed in one of these studies, but not in the other [14, 15]. The relationship between development of SDF and serum lipoproteins components such as CHO, LDL, HDL, VLDL and TG in the pathogenesis of dengue infection, and its underlying mechanism requires further studies.

Notably, DEVN-2 was the cause of the 2015 epidemic while previously only DEVN-1 had been reported in this region. Earlier studies have shown that secondary infection with different DENV serotypes increases the risk of developing SDF [16, 17]. In the case of a new circulating serotype, individuals may be more susceptible to infection and may progess to a serious condition that requires further public health attention.

Our molecular epidemiological study showed that the DENV-2 isolated in Chaozhou belongs to the cosmopolitan genotype, clustering with those from Southeast Asia countries. These findings indicate that southern China is closely linked to Southeast Asia DENV transmission. In fact, there are frequent occurrences of DENV-1 and DENV-2 in Southeast Asian countries [4] whereas the DENV-2 cosmopolitan genotype has also spread to the Indian subcontinent, the South Pacific islands, Latin America, and Somalia [18]. Previous studies have shown that separate groups exist within the Cosmopolitan Genotype [19]. Future phylogenetic analyses based on entire genome sequences are needed to provide more robust data on the nature of Chinese endemicity.

Interestingly, we observed that the strain from this outbreak were closely related to the strains observed in Malaysia from 2010 and 2014, but not to the strains from China in the same years. The phylogenetic analysis was supported by the finding that the first confirmed dengue infected patient in Chaozhou had visited Malaysia early that year. It is possible, therefore, that this year epidemic in Chaozhou may have been caused by the import of viruses from Southeast Asia followed by subsequent local transmission.

In conclusion, our results showed the general clinical features from the patients infected by the dengue virus during the 2015 dengue outbreak in Chaozhou, Guangdong province of China. Many abnormal laboratory indices have been observed in DF patients. A comprehensive analysis of these data could assist and guide the clinical diagnosis and treatment, which is needed for the control of dengue virus infection. In addition, the origins and biological properties of these DENV isolates indicated that the epidemic might have been caused by the importation of viruses from Southeast Asia and subsequent local transmission. China may thus be facing a substantial dengue threat with potential invasion into wider areas and it seems likely that the etiologic DENV strain may persist in Guangdong for a long time. Whether southern China is changing from a dengue epidemic area to an endemic DENV transmission area will require continued clinical observation.
